# HIV-1 Structural Proteins or Cell-Signaling Factors? That Is the Question!

**DOI:** 10.3390/cimb46060306

**Published:** 2024-05-22

**Authors:** Michele Pellegrino, Francesca Giordano, Francesca De Amicis, Maria Marra, Paola Tucci, Stefania Marsico, Stefano Aquaro

**Affiliations:** Department of Pharmacy, Health and Nutritional Sciences, University of Calabria, 87036 Arcavacata di Rende, Italy; michele.pellegrino@unical.it (M.P.);

**Keywords:** HIV-1, HIV structural proteins, virokine

## Abstract

The biological activity of structural HIV-1 proteins is not limited to ensuring a productive viral infection but also interferes with cellular homeostasis through intra- and extracellular signaling activation. This interference induces genomic instability, increases the lifespan of the infected cell by inhibiting apoptosis, and subverts cell senescence, resulting in unrestricted cell proliferation. HIV structural proteins are present in a soluble form in the lymphoid tissues and blood of infected individuals, even without active viral replication. The HIV matrix protein p17, the envelope glycoprotein gp120, the transenvelope protein gp41, and the capsid protein p24 interact with immune cells and deregulate the biological activity of the immune system. The biological activity of HIV structural proteins is also demonstrated in endothelial cells and some tumor cell lines, confirming the ability of viral proteins to promote cell proliferation and cancer progression, even in the absence of active viral replication. This review corroborates the hypothesis that HIV structural proteins, by interacting with different cell types, contribute to creating a microenvironment that is favorable to the evolution of cancerous pathologies not classically related to AIDS.

## 1. Introduction

The introduction of highly active antiretroviral therapy (HAART) has significantly reduced the incidence of HIV-associated disorders, but HIV-infected people remain at increased risk of many types of cancer [1]. Certainly, immunosuppression remains an important risk factor, but the oncogenic properties of HIV could also be attributed to viral proteins’ ability to induce malignant cell transformation and to interfere with multiple homeostatic cellular processes. Indeed, patients living with HIV-1 show chronic immune activation and an inflammatory state. This includes B cell activation and increased T cell turnover, leading to elevated levels of cytokines, chemokines [2] and other inflammatory biomarkers [3]. This chronic inflammatory state is associated with the rapid onset of serious pathologies apparently unrelated to AIDS, such as metabolic syndrome [4,5], coronary heart disease with thrombotic events [6] and neurological disorders [7]. Although AIDS-related cancers have decreased since the advent of HAART, the incidence of lymphomas remains elevated among individuals with chronic HIV-1 infection, with non-Hodgkin lymphoma (NHL) being the predominant type [8]. This indicates that HAART alone, even when effectively suppressing viral replication, does not completely alleviate all the HIV-1-associated complications.

The most credible hypothesis is that circulating viral proteins may contribute to disease progression in patients where the virus is undetectable and in the absence of active HIV-1 replication. In HIV-1 infection, both regulatory and structural proteins, including Tat, Nef, gp120, and p17, can be produced and released from latently infected cells [9,10]. To further regulate many essential functions of the HIV-1 replication cycle (i.e., entry, assembly, budding), the two major structural polyproteins of the retrovirus, Gag and Env, are able to induce signal transduction and subsequent cell activation. This biological activity is essential for efficient HIV-1 infection, because these intracellular and extracellular signals induce genomic instability, which increases the lifespan of infected cells (by inhibiting apoptosis and reversing cellular aging), leading to unlimited cell proliferation. For example, chronic stimulation of B lymphocytes by HIV appears to be essential for the lymphoproliferative role of HIV-1 [11,12,13]. Numerous studies suggest that several HIV proteins, including gp120 [14], Tat [15], Nef [16] and p17 [17,18], mediate the lymphocyte deregulation that precedes lymphoma in HIV patients. HIV-1 structural proteins like gp120 and p17 persist in patients under HAART [19]. Moreover, the increased expression of p17, gp120 and Nef in HIV transgenic mice that develop lymphoma suggests a lymphomagenic role for these proteins [20]. Can HIV viral proteins interact with non-immune human cells? In addition, when HIV replication is blocked by HAART, viral signaling molecules remain among the tools used by HIV-1 to fuel disease progression. In this review, we highlight some representative examples of studies on the signaling capacity of HIV-1 structural proteins in human cells (immune, endothelial, and cancer cells), supporting the idea that these are the microenvironmental proteins most favorable for promoting cell proliferation. This can lead not only to the amplification of the viral infection but also to an increased risk of cancer in HIV-positive patients.

## 2. Gp120: Viral Receptor and/or Viral Chemokine?

Gp120 is a necessary glycoprotein for viral infection, aiding in the penetration of HIV into the host cells [21]. The initial step in HIV-1 infection occurs through the binding of the viral envelope glycoprotein gp120 to CD4, followed by interaction with coreceptors on both T lymphocytes and macrophages [22]. Monocytes/macrophages contribute considerably to the pathogenesis of AIDS because, in addition to rapidly spreading the virus to other cells, they can induce apoptosis in T lymphocytes, astrocytes and neurons [23]. The two coreceptors involved in the infection are CXCR4 (chemokine receptor 4 α) and CCR5 (chemokine receptor 5 β), which play crucial roles in the evolution of infection. HIV-1 strains are phenotypically differentiated according to their ability to use CCR5 and/or CXCR4 based on the binding affinity directly created with gp120 [24]. The binding to the two coreceptors is made possible by the presence of certain amino acids of gp120; in particular, the main determinant of binding is found within the V3 domain, which provides a higher affinity for CCR5 or CXCR4 and thus determines viral tropism [24,25]. The binding of the HIV envelope protein to the chemokine coreceptors (CXCR4, CCR5) mediates two major biological functions: membrane fusion and signal transduction. Besides facilitating viral entry, p120 plays a significant role in AIDS pathogenesis. The interaction of HIV envelope proteins with the chemokine coreceptors CXCR4 and CCR5 is crucial for both membrane fusion and signal transduction processes. Moreover, it is becoming increasingly evident that gp120 plays a role in the development of AIDS, beyond just facilitating viral entry. The binding of viral envelope proteins to these coreceptors activates various intracellular signaling pathways, resembling the signaling induced by chemokines binding to their receptors. This activation leads to different replication rates in macrophages and lymphocytes [26,27], as well as to the ability of certain HIV strains that utilize CXCR4 to induce apoptosis in macrophages [24,28].

**Figure 1:**
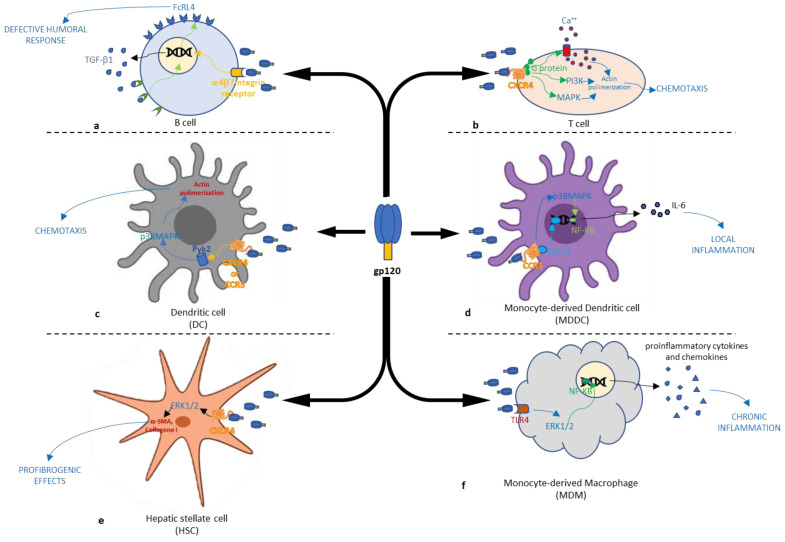
Schematic representation of the signaling pathways involved in the cellular alteration action induced by the HIV-1 gp120 protein: (**a**) interaction with the integrin receptor on B cells causes a defective humoral response; interaction with CXCR4 on T cells (**b**) and on dendritic cells (**c**) causes a chemotaxis increase; interaction with CCR5 on monocyte-derived dendritic cells (**d**) and on monocyte-derived macrophage (**f**) increases inflammation; interaction with CXCR4 on hepatic stellate (**e**) cells causes a profibrogenic effect.

The binding of the HIV-1 envelope glycoprotein gp120 to the chemokine receptor CXCR4 initiates a partial or abnormal array of signals (see Table 1). Within unstimulated human primary CD4+ T cells, the signaling responses triggered by gp120 through CXCR4 closely mimic those induced by the natural ligand, stromal cell-derived factor 1. This activation of gp120 involves heterotrimeric G proteins and major G protein-dependent pathways, leading to events such as calcium mobilization, phosphoinositide-3 kinase activation, and Erk-1/2 MAPK activation. These cascading signals result in swift rearrangements of the actin cytoskeleton and extensive membrane ruffling (Figure 1b). Thus, gp120, in an oligomeric virion-associated form, as well as a monomeric form, elicits a complex cellular response that mimics the effects of a chemokine [29].

Moreover, gp120 is known to be secreted by chronically infected cells [30]. A subset of people living with human immunodeficiency virus demonstrate persistent circulation of gp120 in plasma [31], in saliva [32] and in lymphoid tissues in significant amounts [33]. This finding has important implications for the involvement of gp120 nonentry function and the potential chronicity of the immune deregulation induced, even though virus replication is inhibited by HAART. The binding of gp120 to HIV coreceptors has been documented to elicit diverse effects on dendritic cells (DCs), including the activation of the Pyk2, p38 MAPK, and ERK1/2 signaling pathways (see Figure 1c) [34,35]. Some of these pathways’ activation has been associated with the gp120-induced migration of DCs [34,36].

Furthermore, the in vitro interactions of gp120 with human monocyte-derived dendritic cells (MDDCs) result in atypical maturation and functional changes in these cells, leading to diminished secretion of IL-12 and a decline in allostimulatory capacity [37]. Consistent with these findings, other studies have reported modified expression of surface markers and cytokine secretion in MDDCs following exposure to gp120. The exposure of MDDCs to gp120 results in the inhibition of co-cultured CD4+ T cell proliferation and a decrease in their IL-12 expression. These effects stem from the mannose-dependent interaction between gp120 and a mannose C-type lectin receptor (MCLR), although they are not necessarily associated with the expression of IL-10 [35].

A study proposed carbohydrate-binding agents (CBAs) as novel anti-HIV compounds because they target gp120 glycans, inhibiting infection in primary human monocyte-derived macrophages (MDMs) and effectively preventing MDM-directed viral capture and subsequent transmission to CD4+ T lymphocytes. This is particularly important in transmission through sexual intercourse, since HIV mainly comes into contact with immature DCs present in the vaginal mucosa but also with primary monocyte-derived macrophages (MDM) [38]. Targeting gp120 glycans with CBAs presents a potential avenue for developing anti-HIV compounds, particularly in preventing viral transmission through macrophages.

Del Cornò et al. proposed a model through which gp120 is able to directly activate the STAT-3 and p38 MAPK/NF-ĸB signaling pathways following interaction with CCR5. Precisely, the activation of these two pathways would determine the production of IL-6, a molecule that plays a key role in gp120-mediated STAT-3 activation (Figure 1d). It is important to note that this pathway is exclusively activated by gp120 and not by the natural chemokine ligand CCL4, indicating that gp120 has specific signaling properties that distinguish it from other ligands. In addition, the gp120-driven signaling pathway specifically triggers the activation of the STAT3/IL-6 axis and increases the expression of negative regulators of STAT3 activation, such as PIAS3 [39].

Gp120’s ability to activate specific signaling pathways highlights its impact on cellular processes, cytokine production, and inflammation, influencing various cell types. The interaction between gp120 and TLR4 triggers the activation of the NF-ĸB and MAPK pathways.

**Table 1:** Biological activity of HIV-1 proteins on different cell types.

HIV-1 Proteins	Cells	Signaling	Effects	Ref.
gp120	Human primary CD4+ T cells	Heterotrimeric G protein-dependent pathways, calcium mobilization, PI3K, Erk-1/2 MAPK activation	Chemotaxis	[29]
	Dendritic cells (DCs)	Pyk2, p38 MAPK activation	Migration and inhibition of autophagy	[34]
	Human monocyte-derived dendritic cells (MDDCs)	STAT3/p38 MAPK/NF-ĸB activation	Persistent local inflammation and immune activation (IL-6 secretion)	[39]
	Monocyte-derived macrophages (MDMs)	NF-ĸB and MAPK pathways activation	Local amplification and maintenance of chronic inflammation and infection	[24,40]
	Hepatic stellate cells (HSCs)	ERK 1/2 pathway activation	Profibrogenic effects	[41]
	Primary B cells	Releasing TGF-β1 upregulates FcRL4	Blunt and delay the humoral immune response	[42]
gp41	Human T cell line (Jurkat)	Inhibition of protein kinase C (PKC) activity and calcium mobilization	Inhibits the T cells’ activation	[43]
	Human CD4+ T cells	Caspase-3 activation followed by activation of Bid	Apoptosis	[44]
	Peripheral blood mononuclear cells (PBMCs)	ERK/MAPK pathway activation	Cell proliferation	[45]
p17	Primary human monocytes	AP-1 transcription factor activation	Inflammation (MCP-1 secretion)	[46]
	Plasmacytoid dendritic cells (pDCs)	Down-regulation of nucleophosmin, heat shock protein 70 and eukaryotic translation initiation factor 5B	Cell survival, resistance to apoptotic stimuli, increased proliferation	[47]
	Human T cells	Obg-like ATPase 1 (OLA1) and hyperactivation of glycogen synthase kinase-3 beta (GSK3β)	Autophagy inhibition	[48]
	Human B cells	PTEN/PI3K/Akt pathway activation	Apoptosis inhibition, cell cycle promotion and cancer progression	[18,49]
	Breast cancer cells line (MDA-MB231)	ERK/MAPK pathway activation	Enhance cell migration and invasiveness	[50]
	Human endothelial cells (ECs)	PI3K/Akt and MEK/ERK1/2	Angiogenic activity	[51]
	Primary human lymph node–derived lymphoid endothelial cells (LN-LECs)	PI3K/Akt and MEK/ERK1/2	Lymphangiogenic activity	[52]
p24	Dendritic cells (DCs)	STAT1 and IRF3 pathways activation	Inflammation (INF production)	[53]
	Leukemic monocyte cell line (THP-1)	Enhanced TRIM5α E3 stimulates AP-1 and NF-ĸB signaling via the TAK1/TAB2/TAB3 complex pathway	Inflammation	[54]

This activation, depicted in Figure 1f, leads to the upregulation of proinflammatory cytokines and chemokines in monocyte-derived macrophages (MDMs). In hepatic stellate cells (HSCs), this process leads to cell migration and the secretion of CCL2 and CXCL8, as shown in Figure 1e [40]. Several studies have shown that gp120 stimulates HSC migration, smooth muscle actin expression, and the secretion of procollagen type I and various cytokines involved in the fibrotic process [41,55].

Furthermore, gp120 triggers the production of CCL2 by human macrophages [56] and HSCs, which contributes to the local amplification and persistence of chronic inflammation [57]. Moreover, chemokines like CCL2 and CCL4 act as chemoattractants for HSCs [58], suggesting that gp120 may lead to HSC accumulation through direct chemotaxis [55]. HSCs themselves, along with activated macrophages, release chemokines, further contributing to this accumulation. HSCs and activated macrophages also secrete chemokines, further promoting this accumulation. Additionally, the release of CXCL8 is linked to the advancement of chronic liver disease, with monocytes/macrophages being the main responders to CXCL8 in cirrhosis [59]. Other macrophage-derived inflammatory factors, such as IL-1b, support the survival of activated HSCs [60]. In the absence of infection, gp120 can induce activation and stimulate the expression of collagen I in HSCs through its interaction with CXCR4, which is present on HSCs, thereby triggering profibrogenic effects. The ERK 1/2 pathway is important for collagen I induction in activated HSCs [41].

HIV-1 gp120 binds directly to integrinα4β7 on primary B cells, leading to certain abnormalities in the B cell phenotype and function that are seen in HIV-1 viremic individuals. It is reasonable to assume that some aspects of the defective humoral response in HIV-infected individuals [61] result from a direct interaction between free gp120 and free B cells, which may directly attenuation the activation and proliferation of naive B cells by TGF-β1 release, resulting in an upregulating of the inhibitory IgA receptor FcRL4 (Figure 1a) [42]. The increased expression of FcRL4 caused by gp120 may explain the insufficient mucosal IgA responses observed in acute HIV-1 and SIV infection [62,63,64].

In HPV-positive neoplastic genital and oral epithelial cells, the presence of the HIV-1 gp120 and tat proteins induces the epithelial–mesenchymal transition (EMT), and consequently, tumor invasiveness. Particularly, the interaction between HIV-1 proteins and epithelial neoplastic cells stimulates the expression of stem markers, such as CD133 and CD44. As a consequence, the differentiation of neoplastic cells into cancer stem cells occurs, which causes resistance to therapeutic treatments and promotes tumor invasiveness. The cancer-associated EMT is regulated by the TGF-beta signaling pathway, which is in turn activated by MAPK. Lien et al. demonstrated that inhibition of TGF-beta and MAPK signaling, suppression of the mesenchymal protein vimentin and restoration of E-cadherin, an epithelial protein, reduce the HIV-mediated invasiveness of neoplastic epithelial cells [65]. In summary, gp120’s role in HIV infection extends beyond viral entry, influencing various cellular processes and contributing to the complexity of HIV pathogenesis. The inflammatory pathways STAT3, NF-κB, and PI3K/AKT, activated by gp120 in cells of the immune system, directly or indirectly alter the microenvironment, producing a tumor microenvironment and favoring the development of lymphomas [66,67].

Furthermore, the alteration in the activity of immune cells caused by the HIV-1 glycoprotein could delay and blunt the immune response against cancer cells and favor the evolution of cancer in HIV-positive patients.

Understanding these multifaceted interactions is crucial for developing comprehensive therapeutic strategies.

## 3. Gp41: Viral Transenvelope Protein and/or Cell Signaling Molecule?

The gp41 transmembrane subunit of the HIV-1 envelope glycoprotein (Env) plays a central role in syncytium formation and HIV infection [68]. Its extracellular domain contains four major regions, including the fusion peptide, the N-terminal seven-repeat, the loop, and the C-terminal seven-repeat. The gp41 subunit is divided by the transmembrane region into an endodomain and an ectodomain, with the latter possessing a hydrophobic N-terminal fusion peptide, followed by an N-terminal and C-terminal leucine/isoleucine sequence with a helical structure called HR1 and HR2 [69]. Gp41 also contains consensus sites (aspragin-X-serine/threonine) for the incorporation of N-linked carbohydrates; their presence reduces the binding surface area of gp41, which serves as an immunogenic target [70]. The gp41 trimer is situated in the virus membrane and covered by the gp120 surface protein [71]. Initially, gp120 binds to the CD4 cell surface and a chemokine receptor (CCR5/CXCR4), causing a structural change in the complex and exposing gp41. Then, the gp41 trimer ejects and inserts its fusion peptide into the target cell membrane. Following this, the N-terminal heptad repeats and the C-terminal heptad repeats of gp41 are rearranged to create a stable six-helix bundle that brings the membranes of both the virus and the cells into close proximity to finally accomplish the fusion process and target cells’ infection [72]. As has been shown in the previous paragraph, the interaction of gp120 with the cell surface CD4 receptor and coreceptor CCR5 or CXCR4 triggers a series of cellular signals that support viral infection, while the HIV-1 gp41 can also interact with cell surface proteins to activate cell signals that regulate both viral infection and host cell functions. For instance, gp41 is functionally similar to human type I interferons, and the comparison with interferons suggests that gp41 might contribute to immune modulation beyond its direct involvement in viral entry (Table 1).

**Figure 2:**
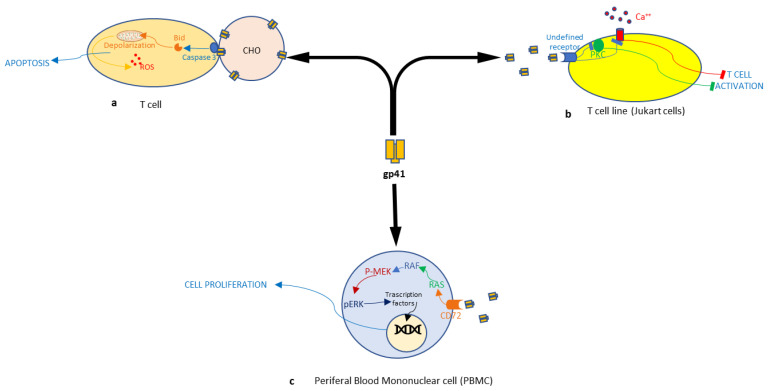
Schematic representation of the signaling pathways involved in the cellular alteration action induced by the HIV-1 gp41 protein: (**a**) the fusion/hemifusion process mediated by gp41 causes human CD4+ T cells’ apoptosis; (**b**) interaction with an undefined receptor blocks Jurkat cells’ activation; (**c**) interaction with CD72 causes peripheral blood mononuclear cells’ proliferation.

Recombinant soluble GP41 can selectively enhance the surface expression of MHC class I, II and ICAM-1 on human T cells, B cells and monocytic cells [73], as well as inhibiting spontaneous proliferation of human cell lines, including the T helper cell line (H9), B cell line (Raji) and monocytic cell line (U937) [74,75]. Indeed, Rueg and Strand have demonstrated that a short sequence of amino acids 539 to 684 from the transmembrane protein gp41 of HIV-1 inhibits the activation of the lymphocytes T cell line (Jurkat) (Figure 2b). This peptide hinders both the influx of intracellular calcium and the enzymatic activity of PKC, which are two key intracellular signal transducers in the phosphoinositide hydrolysis pathway responsible for T-cell activation [43]. The detailed mechanism sheds light on how gp41 might interfere with intracellular signaling, potentially contributing to immunosuppression.

Garg et al. have shown that gp41 is involved in the induction of CD4 T cell apoptosis through a process dependent on caspase-3 activation (Figure 2a). The interaction of gp41 with cellular membranes generates a signal that activates caspase-3 on the membrane, leading to the activation of pro-apoptotic proteins such as Bid and/or Bax. Although Fas and caspase-8 are not involved, gp41-mediated apoptosis has similarities with the extrinsic apoptosis pathway: gp41-mediated hemifusion most likely induces membrane signaling; caspase-3 activation occurs before mitochondrial depolarization; nelfinavir, which inhibits type II apoptosis but does not inhibit type I Fas-mediated apoptosis also inhibits gp41-mediated apoptosis [44]. The association with the apoptosis pathways suggests a potential mechanism for immune cell depletion in HIV infection. T cells play a critical role in controlling cancer cells. Gp41 inhibition of activation and apoptosis, through PKC and Bid/caspase-3 activation, respectively, produces a dysregulation of T cell activity that could favor the evolution of cancer.

Zhou et al., using a yeast two-hybrid screen, showed that CD74 present on the cell surface is a protein capable of binding to gp41. HIV-1 gp41 can bind to the extracellular domain of CD74, mainly through the gp41 loop (peptide 6358: aa 597–611). This region of the gp41 loop is important because it is involved in the control of mediating virus–host-cell interactions and regulating cellular functions. Using PBMCs as target cells, Zhou et al. observed that peptide 6358 and soluble recombinant gp41 (rsgp41) can stimulate phosphorylation of ERK1/2, leading to activation of the CD74-mediated ERK/MAPK pathway (Figure 2c). Several downstream substrates, including transcription factors and other protein kinases (e.g., c-Jun, c-Myc, and NF-ĸB), can be phosphorylated by ERK/MAPK. The interaction of gp41 or gp41 fragments containing the loop sequence with CD74 highlights its ability to interact with host cell proteins, thereby influencing the signaling pathways important for cell proliferation and HIV infection. The authors hypothesize that CD74 plays a role in the early stages of HIV-1 infection by interacting with the gp41 loop region. However, the observed inhibition of HIV-1 infection upon the silencing of CD74 likely occurs due to the stopping of the CD74-mediated ERK/MAPK pathway [45].

Understanding the diverse functions of gp41 presents opportunities for developing targeted therapies. Inhibiting specific interactions or signaling pathways involving gp41 could be explored to modulate immune responses and impede viral replication.

## 4. p17: Viral Matrix Protein and/or Deregulator Virokine?

In mature virions, the matrix protein p17 is a 132-amino acid polypeptide that forms a protective shell attached to the inner surface of the plasma membrane of the virus [76,77]. Nuclear magnetic resonance and X-ray crystallography techniques have been employed to determine the three-dimensional structure of p17 [78,79]. Each p17 molecule is characterized by five major α-helices and a notably basic platform composed of three β-strands. The matrix protein plays a key role in several steps during virus replication. It plays a role in the initial phases of virus replication, contributing to RNA targeting to the plasma membrane, the incorporation of virions into the envelope, and the assembly of particles during the later stages [80]. p17 is released into the extracellular space from HIV-1-infected cells and can be readily identified in the plasma and tissue specimens of patients, including those who have undergone successful HAART [19]. Extracellularly, p17 has been observed to disrupt the biological activities of various cells directly or indirectly implicated in AIDS pathogenesis. All the activities associated with p17 initiation depend on the interaction between the functional epitope (AT20), located in the N-terminal region (amino acids 11 to 30) of the viral protein, and receptors expressed on various target cells [81].

Recent studies have described the ability of p17 to exert chemokine [46], proangiogenic [51] and lymphangiogenic [52] activities and to deregulate the biological activity of different cells (Table 1). These activities are facilitated through the binding of p17 to CXCR1 and CXCR2, which are the physiological receptors for interleukin-8 (IL-8). Indeed, p17 has been identified to mimic certain biological activities of IL-8 [51,82].

**Figure 3:**
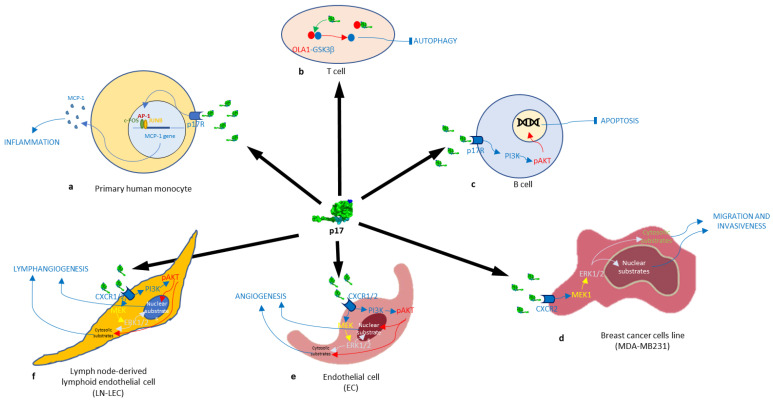
Schematic representation of the signaling pathways involved in the cellular alteration action induced by the HIV-1 p17 protein: (**a**) interaction with p17 receptor on primary human monocytes increases inflammation; (**b**) intracellular interaction with OLA1 inhibits T cell autophagy; (**c**) interaction with p17 receptor inhibits B cells’ apoptosis; (**d**) interaction with CCR2 increases breast cancer cells’ migration and invasiveness; interaction with CXCR1/2 increases angiogenesis (**e**) and lymphangiogenesis (**f**).

Experiments conducted on primary human monocytes have demonstrated that the interaction between p17 and the cellular receptor selectively activates the transcription factor AP-1 (refer to Figure 3a) [82]. More recent data have underscored the Rho/ROCK pathway as a principal target in p17-mediated signaling [17]. The cellular receptor responsible for the chemokine-like activity of p17 on human monocytes is the chemokine receptor CXCR1. Upon binding with CXCR1, p17 induces the chemoattraction of monocytes by activating a signaling pathway that involves Rho/ROCK activation [51]. Rho is recognized for its pivotal role in monocyte migration [83], and its downstream effector ROCK, a serine/threonine kinase, plays a role in the regulation of actin organization [84].

Fiorentini et al. proposed a model in which p17 induces the migration of immature circulating plasmacytoid dendritic cells (pDCs) to the lymph nodes, rendering them incapable of serving as a bridge between the innate and adaptive immune systems. Importantly, p17 has the ability to stimulate circulating pDCs to produce CCR7, which helps in recruiting them to the lymphoid organs. The study observed that p17 can affect host genes, leading to the downregulation of nucleophosmin, heat shock protein 70, and eukaryotic translation initiation factor 5B. Nucleophosmin plays a significant role in enhancing E2F1-mediated transcriptional activity, supporting cell survival, and providing resistance against apoptotic signals. Additionally, the elevated expression of the molecular chaperone hsp70 is associated with increased proliferation and exhibits general cytoprotective properties [47]. The inhibition of apoptosis is not the only mechanism by which the HIV-1 matrix protein alters the biological activities of immune cells, given that it also acts as a major factor in suppressing the autophagic process in immune T cells, especially under glucose starvation conditions. According to Lu et al., HIV p17 interacts with Obg-like ATPase 1 (OLA1), resulting in the hyperactivation of the downstream effector glycogen synthase kinase-3 beta (GSK3β), a critical regulator in the T cell autophagic process (Figure 3b) [48].

Giagulli et al. demonstrated that in B cells, a p17 variant called S75X, derived from a Ugandan HIV-1 strain A1 and differing from the prototype clade B isolate BH10 p17, initiates the activation of the phosphatidylinositol 3-kinase (PI3K)/Akt signaling pathway. Consequently, the observation of S75X revealed an increase in B cell proliferation and clonogenicity on soft agar, serving as preliminary evidence of the existence of a p17 variant with oncogenic activity that specifically targets human B cells [17]. A single mutation in the wild-type p17 protein, replacing Arginine (R) with Glycine (G) at position 76 (p17R76G), similar to the S75X variant, can give B cells the ability to clonally expand. This mutation activates the PTEN/PI3K/Akt signaling pathway and alters the levels of various molecules involved in preventing cell death (such as CASP-9, CASP-7, DFF-45, NPM, YWHAZ, Src, PAX2, MAPK8), promoting cell growth, and advancing cancer (like CDK1, CDK2, CDK8, CHEK1, CHEK2, GSK-3β, NPM, PAK1, PP2C-α) (Figure 3c) [49].

The distinct biological activity of S75X and p17R76G compared to wild-type p17 underscores how the matrix protein’s binding to and signaling through the p17-receptor(s) may involve different determinants of the structure or conformation. The molecular basis for these opposing mechanisms may depend on the exposure or lack thereof of a clonogenic epitope and the activation of the PTEN/PI3K/Akt pathway, which serves as a critical driver of lymphoma development and metastasis. Indeed, Dolcetti et al. [18] demonstrated that p17 (vp17) variants derived from non-Hodgkin lymphoma (NHL) tissues of HIV-positive individuals exhibited potent B cell growth-promoting activity. These variants are distinguished by the amino acid insertion at position 117–118 (Ala-Ala) or 125–126 (Gly–Asn or Gly–Gln–Ala–Asn–Gln–Asn), as well as other mutations scattered throughout the series. Identical dominant vp17s were found in both tumors and plasma. Ala insertion at positions 117–118 promotes B cell growth and activates the Akt cell signaling pathway. Ultradeep pyrosequencing showed that vp17 has a C-terminal insertion found more frequently in the plasma of HIV-positive individuals with non-Hodgkin lymphoma (NHL). The Ala–Ala insertion at position 117–118 in wild-type p17 was found to be sufficient to confer B cell growth-promoting activity. In contrast, p17 with the Gly–Asn insertion at position 125–126 did not exhibit such expression. Physiological analysis indicates that the Ala–Ala insertion mutant is less stable than wild-type p17, whereas the Gly–Asn form is stabilized [18].

Recent data reveal that both the wild-type p17 and certain vp17s isolated from the plasma of HIV+ patients with non-Hodgkin lymphoma (NHL) significantly enhance the migration and invasiveness of MDA-MB-231 breast cancer cells. The heightened aggressiveness of MDA-MB-231 behavior is attributed to the activation of the MAPK pathway following the interaction of the viral protein with CXCR2 (refer to Figure 3d). Indeed, the phosphorylation status of ERK1/2 increased in MDA-MB-231 cells treated with either wild-type p17 or vp17s. Additionally, inhibiting the ERK-dependent pathway with PD98059, targeting the upstream kinase MEK1, significantly decreased the migratory activity induced by p17 in the cells [50]. Moreover, p17, related to IL-8, has a strong binding to CXCR1 and CXCR2 and shows potent angiogenic activity on human endothelial cells (ECs) (Figure 3e). Experimental evidence identifies Akt and ERK as the signaling molecules responsible for p17’s proangiogenic function. Additionally, PI3K plays a crucial role in connecting CXCR1 and CXCR2 to Akt and ERK signaling. The transmission of AKT signals to ERK occurs through MAPK/ERK kinase (MEK), as the MEK inhibitor PD980099 effectively blocks p17-induced capillary-like structure formation [51].

Similar events to those observed in p17-treated endothelial cells (ECs) are triggered in primary human lymph node-derived lymphoid endothelial cells (LN-LECs) following p17 stimulation (refer to Figure 3f). The HIV matrix protein exerts its lymphangiogenic activity through both CXCR1 and CXCR2, triggering the PI3K/Akt. The MEK/ERK1/2 signaling pathways play a crucial role in p17-induced capillary-like structure formation, as demonstrated by the complete blockade of this process with specific inhibitors of both pathways [52]. In conclusion, p17, once primarily considered a structural component of the HIV virion, emerges as a multifunctional protein with diverse activities affecting immune cells, oncogenic potential, and involvement in angiogenesis. Understanding these intricate functions provides valuable insights into potential therapeutic targets and the complex interplay between HIV and host cells.

## 5. p24: The Building Block of the Capsid Core or Proinflammatory Molecule?

The capsid (CA) protein of HIV-1 plays a vital role as an essential structural component of the virion, facilitating various critical steps in the virus life cycle. The Gag polyprotein undergoes cleavage by the viral protease, resulting in the generation of individual mature viral proteins arranged from the N- to C-terminus as a matrix (p17), nucleocapsid (NC), and p6. Additionally, two spacer peptides (SP1 and SP2) and CA are produced in this process [85,86]. In the mature virion, the assembly of CA forms a shell surrounding the viral RNA genome and core-associated proteins and the asymmetric architecture of the fullerene-shaped cone is the result of 12 pentamers and approximately 250 hexamers [87]. Indeed, the capsid protein (CA) plays a pivotal role in various crucial processes throughout the HIV-1 life cycle, contributing significantly to both the early and late stages of the viral replication process. The CA protein is indispensable in virus infection and replication as it offers structural support and protection to both the reverse transcription complex (RTC) and the preintegration complex (PIC). Simultaneously, it enables the diffusion of deoxyribonucleotide triphosphates (dNTPs) for reverse transcription [88]. Additionally, CA plays a crucial role in facilitating retrograde movement in the cytoplasm, nuclear import, and proper localization [89,90]. Kuster et al. proved that in patients with early, asymptomatic HIV infection before and during combination HAART, the presence of viral p24 remained consistently detectable in the tissues, even in cases where the HIV-1 RNA in plasma was fully suppressed. Additionally, there was an increase in viral p24, even following short-term rebounds in the plasma viral load [91].

The CA monomer is a 231-residue long protein, distinguished by 2 prominent α-helical domains: the N-terminal and C-terminal domains, connected by a 5-residue linker spanning 146 to 150. The N-terminal, often referred to as the “central” domain (consisting of residues 1 to 145), plays a role in virion maturation and the binding of the cytosolic protein cyclophilin A (CypA). On the other hand, the C-terminal domain, also known as the “dimerization” domain (residues 151 to 231), contributes to Gag–Gag interactions [92].

**Figure 4:**
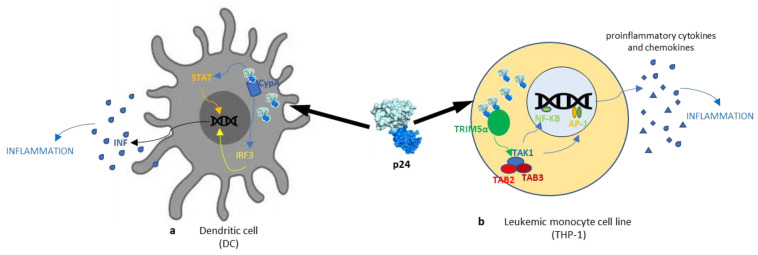
Schematic representation of the signaling pathways involved in the cellular alteration action induced by the HIV-1 p24 protein. Intracellular interaction with cyclophilin A (CypA) in dendritic cells (**a**) activates both STAT and IRF3 INF induction, increasing inflammation. In a leukemic monocyte cell line (**b**), interaction between TRIM5α and hexameric p24 stimulates AP-1 and NF-KB signaling via the TAK1/TAB2/TAB3 complex pathway and elicits proinflammatory signaling pathways.

The interaction between CypA and CA triggers a change in p24 conformation that alters the viral envelope and thereby affects the ability of intracellular sensors to detect viral nucleic acids, which in this case act as a pathogen-associated molecular template (Table 1). Manel et al. found that during late infection, the interaction between the CA domain of Gag and the newly synthesized CypA induces a type I IFN response that activates T cells. The authors also demonstrated that in DCs, the CA–CypA interaction activates both STAT1 and IRF3. The IFN induction and IFN production pathway (Figure 4a) events were specifically abrogated by infecting target cells with the HIV-1 G89V-binding CypA-CA mutant [53].

Pertel et al. demonstrated that the interaction between TRIM5α, the AC-specific restriction factor, and the AC hexameric network stimulates AP-1 and NF-ĸB signaling through the TAK1/TAB2/TAB3 complex (MAP3K7) pathway. They also showed that the HIV-1 CA network enhances the activity of TRIM5α E3 ubiquitin ligase, which activates TAK1 and induces inflammatory genes. Therefore, TRIM5α can be considered a pattern recognition receptor (PRR) that recognizes CA retrovirus and activates inflammatory signaling pathways (Figure 4b) [54]. In summary, the HIV-1 CA protein, traditionally seen as a structural element, plays a dynamic role in multiple aspects of the virus life cycle. Its interactions with cellular factors like CypA and TRIM5α highlight its influence on viral uncoating, innate immune responses, and proinflammatory signaling pathways, underscoring its pivotal role in the host–pathogen interplay during HIV infection.

## 6. Conclusions

The HIV “accessory proteins” (Nef, Vif, Vpr, and Vpu or Vpx in HIV-2) enhance the efficiency of viral replication through interactions with human cells. For example, Nef negatively regulates CD4 by promoting its endocytosis and lysosomal degradation. This facilitates virus budding by removing the Env receptor from the cell surface. Nef also reduces the expression of major histocompatibility complex (MHC) class I on cell surfaces, thereby limiting the ability of the immune system to eliminate infected cells. Additionally, Nef activates T cells by binding to the T cell receptor and several downstream effectors. Activated T cells translocate the transcription factors NFAT and NF-ĸB into the nucleus, where they are thought to serve as viral promoters, leading to increased HIV transcription. This is just one example of the biological power of the HIV accessory proteins. While the pathogenic action of the HIV accessory proteins linked to an amplification of the infection is widely recognized, it is necessary to study in more depth the ability of the HIV structural proteins to interact with human cells, modulating their metabolism, the progression of cell cycle, differentiation capacity, motility and genomic stability. The HIV structural proteins not only constitute the virion, but even in the absence of productive infection, they are always detectable in the HIV-positive patient and are capable of triggering aberrant molecular mechanisms and signaling pathways in human cells. From this review, it emerges that HIV structural proteins contribute to the pathogenesis of the infection by interacting with the cells of the immune system (Table 1). However, through cell signaling, the HIV structural proteins can also trigger malignant transformation of normal cells, as well as proliferation and dissemination of existing precancerous and cancerous cells. At the same time, it increases the growth and metastatic activity of tumors (Table 1).

Further experimental studies are needed to confirm the activation of signal transduction pathways in different cell types by the HIV-1 structural proteins, as many examples reported in this review are present as single studies. Furthermore, further clinical studies on patients undergoing HAART treatment would be appropriate to confirm both the presence of individual viral proteins in serum and any pathogenic effects on organs and tissues.

A better understanding of the dynamic interactions between the HIV-1 structural proteins and the target cells may lead to the development of antiviral strategies that can limit HIV-1 pathogenesis. Thus, the significant importance of blocking the biological activity of the HIV structural proteins becomes clear. This can be achieved using specific drugs or vaccines that induce neutralizing antibody responses against these viral proteins to prevent and combat AIDS-related pathologies.

## Data Availability

Not applicable.
